# Qualitative Heterogeneous Signal Drop on Chemical Shift (CS) MR Imaging: Correlative Quantitative Analysis between CS Signal Intensity Index and Contrast Washout Parameters Using T1-Weighted Sequences

**DOI:** 10.3390/tomography7040079

**Published:** 2021-12-14

**Authors:** Arnaldo Stanzione, Francesco Verde, Roberta Galatola, Valeria Romeo, Raffaele Liuzzi, Pier Paolo Mainenti, Giovanni Aprea, Michele Klain, Elia Guadagno, Marialaura Del Basso De Caro, Simone Maurea

**Affiliations:** 1 Department of Advanced Biomedical Sciences, University of Naples “Federico II”, 80131 Naples, Italy; arnaldo.stanzione@unina.it (A.S.); francesco.verde2@unina.it (F.V.); valeria.romeo@unina.it (V.R.); michele.klain@unina.it (M.K.); elia.guadagno@unina.it (E.G.); marialaura.delbassodecaro@unina.it (M.D.B.D.C.); maurea@unina.it (S.M.); 2 Institute of Biostructures and Bioimaging, The National Research Council (CNR), 80131 Naples, Italy; raffaele.liuzzi@cnr.it (R.L.); pierpaolo.mainenti@ibb.cnr.it (P.P.M.); 3 Department of Clinical Medicine and Surgery, University of Naples “Federico II”, 80131 Naples, Italy; giovanni.aprea@unina.it

**Keywords:** adrenal tumors, MRI, quantitative imaging, chemical shift, dynamic post-contrast sequence

## Abstract

The aim of this study was to calculate MRI quantitative parameters extracted from chemical-shift (CS) and dynamic contrast-enhanced (DCE) T1-weighted (T1-WS) images of adrenal lesions (AL) with qualitative heterogeneous signal drop on CS T1-WS and compare them to those of AL with homogeneous or no signal drop on CS T1-WS. On 3 T MRI, 65 patients with a total of 72 AL were studied. CS images were qualitatively assessed for grouping AL as showing homogeneous (Group 1, *n* = 19), heterogeneous (Group 2, *n* = 23), and no (Group 3, *n* = 30) signal drop. Histopathology or follow-up data served as reference standard to classify AL. ROIs were drawn both on CS and DCE images to obtain adrenal CS signal intensity index (ASII), absolute (AWO), and relative washout (RWO) values. Quantitative parameters (QP) were compared with ANOVA analysis and post hoc Dunn’s test. The performance of QP to classify AL was assessed with receiver operating characteristic analysis. CS ASII values were significantly different among the three groups (*p* < 0.001) with median values of 71%, 53%, and 3%, respectively. AWO/RWO values were similar in Groups 1 (adenomas) and 2 (benign AL) but significantly (*p* < 0.001) lower in Group 3 (20 benign AL and 10 malignant AL). With cut-offs, respectively, of 60% (Group 1 vs. 2), 20% (Group 2 vs. 3), and 37% (Group 1 vs. 3), CS ASII showed areas under the curve of 0.85, 0.96, and 0.93 for the classification of AL, overall higher than AWO/RWO. In conclusion, AL with qualitative heterogeneous signal drop at CS represent benign AL with QP by DCE sequence similar to those of AL with homogeneous signal drop at CS, but different to those of AL with no signal drop at CS; ASII seems to be the only quantitative parameter able to differentiate AL among the three different groups.

## 1. Introduction

In adrenal imaging, T1-weighted chemical shift (CS) sequence using magnetic resonance imaging (MRI) has shown to be effective to characterize adrenal lesions (AL) and, particularly, to identify adrenal adenomas. Its diagnostic imaging ability depends on the presence of a large amount of intracytoplasmic lipids in adrenal adenomas cells which reflects in homogeneous signal drop on CS out-of-phase images. As a rule, these adenomas are defined as “lipid-rich” [1,2,3]. On the other hand, when the tumor cells do not contain a substantial amount of cytoplasmatic lipids, no signal drop on CS out-of-phase images occurs, and usually these lesions are defined as non-adenomas [1,2,3]. However, adrenal adenomas with low fat within their cytoplasm (“lipid-poor”) do not show signal drop on CS imaging [4]. Moreover, malignant adrenal tumors, such as primary carcinoma or metastases by renal cell (RCC) and hepatocellular (HCC) carcinomas, may contain small amounts of intracytoplasmic lipids, resulting in heterogeneous signal drop on CS sequence [4]. In addition, a heterogeneous pattern of signal drop on CS MRI has also been previously described in benign AL [5]. In particular, this qualitative study demonstrated areas of signal drop intermingled with areas of no suppression in such lesions [5]. Furthermore, heterogeneity of signal drop on CS MRI has been also reported in collision adrenal tumors, and CS artifact (“India-ink”) may mimic signal drop in small adrenal nodules, hence causing additional interpretative imaging pitfalls, and may affect the overall diagnostic performance [6]. 

The added value of quantitative indices over the qualitative assessment of AL behavior on CS images has been already assessed in most studies without showing a diagnostic improvement [3]. Recently, the relation between the signal drop on chemical-shift T1-weighted sequence and the fat fraction measured by mDIXON-Quant sequence for characterizing adrenal lesions has been investigated, demonstrating a similar diagnostic accuracy [7]. However, only few studies investigated the match between qualitative and quantitative assessment of signal drop on CS images, and the majority was conducted at 1.5 T [1]. Finally, heterogeneity of MRI signal intensity for AL characterization has been also described on T2-weighted images. Recently, Tu et al. suggested that the combined qualitative evaluation of T2-weighted signal intensity and heterogeneity was highly accurate in differentiating adrenal metastases from benign, lipid-poor adrenal adenomas [8], while there was no difference in these qualitative parameters comparing lipid-poor and lipid-rich adrenal adenomas [9].

Dynamic contrast-enhanced (DCE) MRI sequence has also been proposed as a further imaging modality to characterize benign AL, mainly adenomas with homogeneous signal drop, showing the wash-out rate as a reliable parameter. Indeed, such did demonstrate early contrast enhancement with significant wash-out on delayed imaging [10,11,12,13]. Conversely, non-adenoma adrenal tumors with no signal drop on CS, such as pheochromocytoma or malignant neoplasms, tend to exhibit a significantly lower wash-out pattern [10,11,12]. However, a significant overlap in DCE MRI parameters has been reported between pheochromocytomas and adenomas [14]. Furthermore, we recently observed that “lipid-poor” adrenal adenomas, with no signal drop on CS, may have a similar wash-out pattern compared with non-adenoma solid adrenal tumors [13]. To our knowledge, no studies have been performed to investigate the DCE behavior of AL showing qualitative heterogeneous CS signal drop.

The aim of this study was to calculate MRI quantitative parameters, extracted from CS and DCE T1-weighted sequences, of AL with qualitative heterogeneous signal drop on CS T1-weighted sequence and compare them to those of AL with homogeneous, or without, signal drop on CS.

## 2. Materials and Methods

### 2.1. Patient Population

This retrospective study was approved by our Institutional Review Board and informed consent waived (PG/2021/0034768, 7 April 2021). Our institutional database was searched to identify abdominal MRI scans performed to characterize AL between 2008 and 2018. Consecutive patients were included in the analysis when meeting the following criteria: (1) imaging protocol including both CS and DCE imaging (with a delayed acquisition at 5 and 10 min); (2) availability of either histopathological results or clinical-imaging follow-up (≥12 months) to serve as reference standard; (3) nuclear medicine imaging studies, such as ^18^F-fluorodeoxyglucose (FDG) PET/CT or ^131^I-metaiodobenzylguanidine (MIBG) scintigraphy, were retrieved whenever available to confirm diagnosis in patients without histopathological results. Patients were excluded in case of artifacts significantly affecting image quality.

### 2.2. Imaging Protocol

MR images were obtained on a 3 T scanner (Magnetom Trio, Siemens, Germany), using a surface-body coil. MR imaging protocol included the following unenhanced sequences: T1-VIBE (TR/TE = 4.04/1.26 ms; TR/TE = 4.04/2.59; slice thickness = 3 mm; no gap) in- and out-of-phase on axial planes, T2 HASTE (TR/TE = 2000/90 ms; slice thickness = 3 mm; gap = 0.6 mm) with fat suppression on axial planes, T2 HASTE (TR/TE = 2000/90 ms; slice thickness = 3 mm; gap = 0.6 mm) on axial and coronal planes. Dynamic contrast-enhanced (0.1 mmol/kg Gd-DTPA Magnevist, Bayer Pharma, Berlin, Germany) T1 VIBE 3D breath-hold sequences (TR/TE = 3.3/1.1 ms; slice thickness = 2 mm; no gap) were acquired before and after contrast injection at arterial (30 s), portal (60 s), and delayed (5 min and 10 min) phases on axial planes.

### 2.3. Image Analysis

Working blinded to clinical data and in consensus, two experienced radiologists reviewed MRI scans of each included patient to identify ALs. In case of disagreement, a third senior radiologist was consulted to reach final agreement. Subsequently, they were asked to classify ALs in three groups based on the CS images findings, as reported in a previous study [5]. AL were visually classified as homogeneous signal drop (Group 1) when a complete signal loss was observed; heterogeneous signal drop (Group 2) when incomplete and inhomogeneous signal loss was found; and no change (Group 3) when no signal loss occurred between in and out phase images. 

Thereafter, the quantitative imaging analysis was performed. Firstly, AL size was recorded in terms of maximum axial diameter (mm). Then, regions of interest (ROIs) were positioned over the detected lesions. In detail, CS and DCE images were manually annotated by drawing a two-dimensional ROI on the slice in which the lesion showed the maximum diameter, to include the entire lesion excluding borders to minimize the possible bias induced by the India-ink artifact. If present, macroscopic areas of fluid collection were excluded, too. On CS images, ROIs were positioned on out-of-phase images and then copied and pasted on the corresponding in-phase images. Similarly, for DCE sequence, ROIs as large as possible were drawn on portal phase images and then copied and pasted on precontrast and delayed (both 5 and 10 min) images. For each lesion, segmentation was performed twice, with the second ROI being placed on a different slice whenever possible. Signal intensity from the two ROIs was then averaged and the mean value was used for the analysis [15]. Image segmentation was performed by the two radiologists working in consensus, with a senior radiologist involved for problem-solving issues. Quantitative CS and DCE parameters were calculated as follows:

1. The percentage of signal intensity reduction on CS sequence was estimated applying the formula of the adrenal signal intensity index (*ASII*) [15]:

$$ASII=\left(\frac{\left(SI_{i}-SI_{o}\right)}{\left(SI_{i}\right)}\right)\times 100$$

where *SI_i_* is the *SI* on in-phase image and *SI_o_* is the signal intensity measured in the out-of-phase image.

2. The percentages of absolute and relative wash-out of AL at 5 and 10 min on DCE images using the following formulas already reported in a previous study [13]:

$$AWO=\left(\frac{\left(SI_{port}-SI_{del}\right)}{\left(SI_{port}-SI_{pre}\right)}\right)\times 100$$

$$RWO=\left(\frac{\left(SI_{port}-SI_{del}\right)}{SI_{port}}\right)\times 100$$

where *SI_port_* is the *SI* measured on portal phase, *SI_del_* is the *SI* measured on delayed phase (5 and 10 min), and *SI_pre_* is the *SI* measured in the precontrast images.

### 2.4. Statistical Analysis

Lesion size, ASII, AWO, and RWO (both at 5 and 10 min) were compared among the three groups of AL. For descriptive statistics, continuous data are expressed as median and range. The nonparametric sum rank test of Kruskal–Wallis and the post hoc Dunn’s test were performed to investigate quantitative parameters differences among the three groups of AL [16,17]. To assess the performance of ASII, AWO, and RWO in discriminating among the three groups, the empirical receiver operating characteristic (ROC) curves, as well as the area under the ROC curve (AUC), were calculated and the optimal cut-off point was determined by Youden’s test [18,19,20]. ROC curves obtained with DCE parameters were then compared to those of ASII [21]. A value of *p* ≤ 0.05 was considered statistically significant. Bias-corrected and accelerated bootstrapping (1000 iterations) was used to calculate 95% confidence intervals [22]. All analyses were performed using the Med-Calc Statistical Software (MedCalc Software, Mariakerke, Belgium).

## 3. Results

### 3.1. Patient Population

Of the 116 consecutive patients retrieved with the institutional archive search, 28 underwent unenhanced MRI scans, while 19 lacked an appropriate reference and were therefore excluded. Similarly, four patients had to be excluded due to the presence of imaging artifacts. Therefore, the final study population included 65 subjects (43 women and 22 men, median age 63 years, age range 23–92 years) and a total of 72 AL (5 bilateral, one patient had 3 lesions). In detail, histopathology results were available for 24 lesions (6 adenomas, 7 pheochromocytomas, 3 myelolipomas, 1 oncocytoma, 5 primary malignant tumors, and 2 metastasis). For the remaining eight non-adenoma lesions (five pheochromocytomas and three metastasis), data from nuclear medicine imaging studies (five MIBG scintigraphy and three FDG PET/CT) confirmed the diagnosis and served as reference standard alongside with clinical-imaging follow-up (≥12 months). Finally, 40 lesions were classified as adenomas as they proved stable in terms of both size and imaging characteristics at imaging follow-up (the mean size of these lesions was 21.3 mm ± 11.3). Based on the qualitative CS evaluation, AL were divided into Group 1 (*n* = 19), Group 2 (*n* = 23), and Group 3 (*n* = 30). A detailed overview of AL distribution in the three groups is reported in Table 1. Of note, a perfect agreement was found between the two radiologists in all cases, without the need to consult the third senior radiologist.

### 3.2. Lesion Size Analysis

No statistically significant differences emerged in AL size among the three groups (*p* = 0.098). In particular, the median value of AL size was 19 mm (ranging from 9 to 37 mm) in Group 1, 29 mm (ranging from 6 to 80 mm) in Group 2, and 24 mm (ranging from 5 to 120 mm) in Group 3.

### 3.3. CS and DCE Quantitative Analysis

Regarding ASII values, they were significantly different in the three groups (*p* < 0.001) with the highest median values found in Group 1 and the lowest in Group 3, while Group 2 showed intermediate values. The analysis of both AWO and RWO showed significant differences between Group 1 and 2 versus Group 3 (*p* < 0.001), while no significant differences in these dynamic parameters were found between Group 1 and 2. In particular, both AWO and RWO values were significantly higher in AL of Group 1 and 2 compared to Group 3, in the evaluation of 5 and 10 min images. Table 2 shows the median values of ASII, AWO, and RWO (both at 5 and 10 min) in the three groups.

### 3.4. ROC Curve Analysis

**Figure 1:**
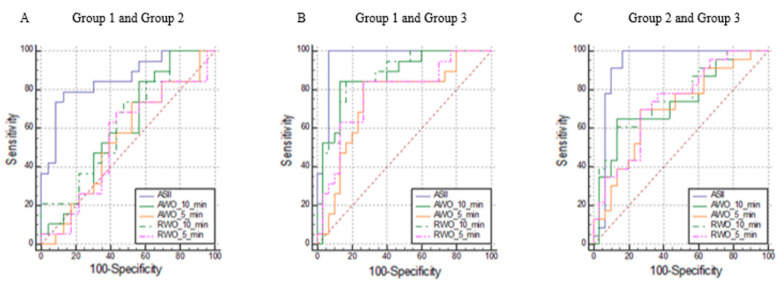
Receiver operating characteristic curves showing the performance of the adrenal signal intensity index (ASII), relative, and absolute washout (RWO and AWO) at 5 and 10 min to differentiate between adrenal lesions of Group 1 and Group 2 (**A**), Group 1 and Group 3 (**B**), and Group 2 and Group 3 (**C**).

Figure 1 illustrates the ROC curves of MRI quantitative parameters in the classification of AL, while in Table 3, the best cut-off value and the corresponding AUCs with 95% confidence intervals are reported. Regarding the ROC curves comparison, ASII showed significantly higher AUCs values compared to AWO and RWO at 5 min in all settings and compared to AWO and RWO at 10 min for Group 1 vs. 2 and 2 vs. 3 (*p* < 0.05). Although ASII was more accurate than AWO and RWO at 10 min in the Group 1 vs. 3 classification, statistical significance was not reached in this case (*p* = 0.09 and *p* = 0.08, respectively).

**Figure 2:**
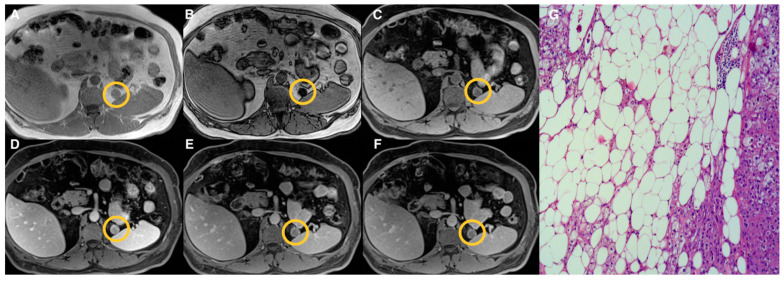
Left “lipid-rich” adrenal adenoma (Group 1) measuring 18 mm (circled in yellow on MR images). T1-VIBE axial scan “in-phase” (**A**) and “out of phase” (**B**), T1-weighted axial dynamic scans with fat suppression before gadolinium administration (**C**), in portal (**D**) and delayed phases at 5 (**E**) and 10 (**F**) minutes; the lesion showed homogeneous signal intensity loss on “out-phase” image (**B**) compared to in-phase image (**A**) and enhancement in the portal phase (**D**) after contrast administration with significant wash-out in delayed phases both at 5 (**E**) and 10 (**F**) minutes. Haematoxylin and eosin stain at 10× magnification (**G**) showed that the lesion was composed of a large amount of fat foci.

**Figure 3:**
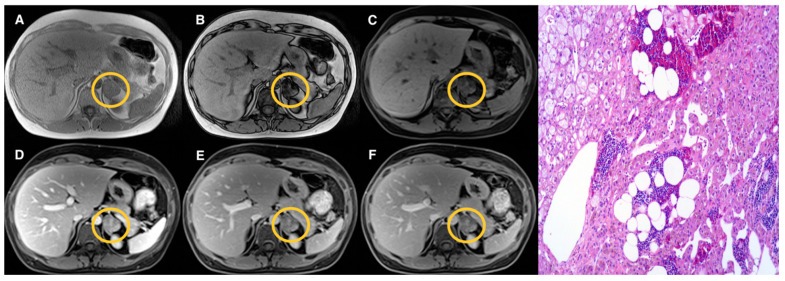
Left adrenal adenoma (Group 2) measuring 33 mm (circled in yellow on MR images). T1-VIBE axial scan “in-phase” (**A**) and “out of phase” (**B**), T1-weighted axial dynamic scans with fat suppression before gadolinium administration (**C**), in portal (**D**) and delayed phases at 5 (**E**) and 10 (**F**) minutes; the lesion showed a heterogenous “patchy” pattern of signal intensity loss on “out-phase” image (**B**) compared to “in-phase” image (**A**); after contrast administration, early enhancement of the lesion on portal phase (**D**) is appreciated with significant wash-out in delayed phases (**E**,**F**) similarly to the lesion of Figure 1 (Group 1). Haematoxylin and eosin stain at 10× magnification (**G**) revealed a small amount of fat foci in the lesion.

**Figure 4:**
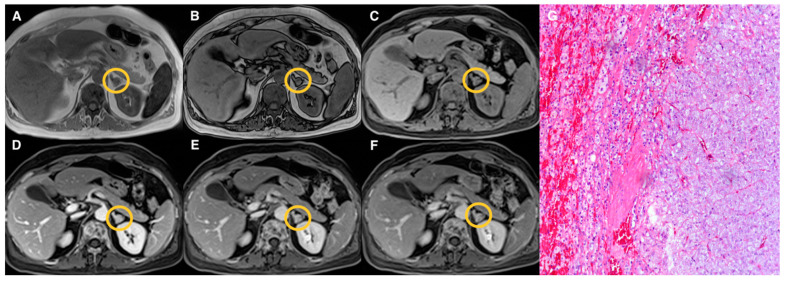
Left “lipid-poor” adrenal adenoma (Group 3) measuring 10 mm (circled in yellow on MR images). T1-VIBE axial scan “in-phase” (**A**) and “out of phase” (**B**), T1-weighted axial dynamic scans with fat suppression before gadolinium administration (**C**), in portal (**D**) and delayed phases at 5 (**E**) and 10 (**F**) minutes; the lesion did not show signal intensity loss on “out-phase” image (**B**) compared to “in-phase” image (**A**); after contrast administration, early and progressive enhancement on portal and delayed phases (**D**–**F**) is appreciated with no significant wash-out. Haematoxylin and eosin stain at 10× magnification (**G**); both cortical (on the left) and medullary (on the right) adrenal tissue were hyperplastic, without evidence of lipomatous changes.

**Figure 5:**
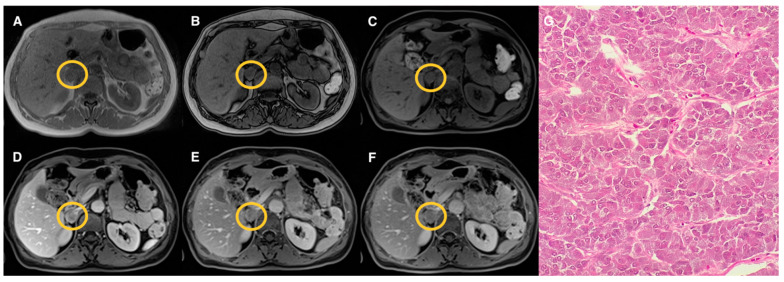
Right pheochromocytoma (Group 3) measuring 21 mm (circled in yellow on MR images). T1-VIBE axial scan “in-phase” (**A**) and “out of phase” (**B**), T1-weighted axial dynamic scans with fat suppression before gadolinium administration (**C**), in portal (**D**) and delayed phases at 5 (**E**) and 10 (**F**) minutes; the lesion did not show signal intensity loss on “out-phase” image (**B**) compared to “in-phase” image (**A**) as the lesion of Figure 3; after contrast administration, early enhancement on portal phase (**D**) is appreciated with no significant wash-out in delayed phases (**E**,**F**). Haematoxylin and eosin stain at 20× magnification (**G**); the neoplasm was composed of nests of cells of medium size and characterized by basophilic cytoplasm and mild nuclear pleomorphism. No lipomatous foci were detected.

Examples of lesions from the three groups are shown in Figure 2, Figure 3, Figure 4 and Figure 5. Specifically, Figure 2 shows an example of a “lipid-rich” adenoma with homogeneous CS signal drop (Group 1) in which a significant wash-out was observed. Figure 3 shows an example of an adrenal adenoma with heterogeneous CS signal drop (Group 2) in which a significant wash-out was observed, similarly to lesions of Group 1. Finally, Figure 4 and Figure 5 show two examples of AL of Group 3 with no CS signal drop and no significant wash-out, respectively, but with different histopathology represented by prevalent “lipid-poor” adenoma (Figure 4) and pheochromocytoma (Figure 5).

## 4. Discussion

The clinical significance of heterogeneous signal drop on CS MRI in AL has not been codified yet [4]. To the best of our knowledge, merely a single experience has previously described and investigated an AL with this CS pattern characterized by a mixed composition of “lipid-rich” and “lipid-poor” cells that exclusively included benign tumors such as hyperplasia or adenomas [5]. However, heterogeneous signal drop on CS sequence may also occur in malignant AL [4]. Thus, heterogeneous signal drop on CS MRI may frequently occur in AL and is challenging for clinical imaging purposes.

Our results indicate that the prevalence of AL with heterogeneous CS signal drop might be higher than what previously reported [5]. Although the sample size of this last study was superior and this discrepancy is probably due to our lower sample size, it can be hypothesized that such AL may occur in clinical practice more frequently than expected. While the pathologic-imaging correlation of these lesions in our study confirmed the mixed composition of “lipid-rich” and “lipid-poor” cells, additional evidence emerged regarding the nature of such lesions. Indeed, six non-adenoma lesions (26%) were found in Group 2. However, they were all benign entities (pheochromocytomas and myelolipomas), in accordance with previous evidence [5]. The CS quantitative assessment confirmed that AL qualitatively classified in Group 2 significantly differ from lesions showing either complete or no signal drop on CS, reflecting a different lipid content. Indeed, this somewhat-expected result of the quantitative analysis of ASII demonstrated significantly different values among the three groups of lesions, showing the highest value in Group 1 and the lower one in Group 3, respectively, corresponding to “lipid-rich” and “lipid-poor” lesions. In Group 2, the value of ASII was intermediate between Groups 1 and 3, suggesting a mixed tumor population of “lipid-rich” and “lipid-poor” cells. These pieces of evidence are further supported by the results of ASII ROC curve analysis, indicating that heterogeneous signal drop in AL is detectable for ASII values between 20% and 60%. Interestingly, ASII was overall superior compared to DCE quantitative parameters in the classification of AL. Finally, we also confirmed that the qualitative assessment of CS signal drop of AL matched the quantitative one on MRI scanners with an higher field strength (3 T), as previously demonstrated at 1.5 T [1].

MRI using DCE sequence has been proposed as an additional modality to differentiate between adenomas and benign, non-adenoma lesions or malignant tumors using the quantitative analysis of wash-out parameters such as RWO and AWO [10,11,12]. In particular, wash-out rates have been demonstrated as reliable parameters to characterize AL, as these lesions show early contrast enhancement with significant wash-out on delayed imaging, while non-adenoma adrenal tumors, such as pheochromocytoma or malignant neoplasms, tend to exhibit significantly lower wash-out values. The results of the present study suggest that AL with heterogeneous signal drop on CS sequence mostly represent benign AL, such as adenomas, as also supported by the values of wash-out quantitative parameters extracted from DCE sequence as well as by clinical-imaging follow-up data or pathology used as standard of reference. Interestingly, we observed similar values of wash-out parameters, both of RWO and AWO, in AL with homogeneous or heterogeneous signal drop on CS. In particular, the percentage values of both RWO and AWO were indicative of benign adrenal tumors either in case of lesions with homogeneous or heterogeneous CS signal drop with no significant difference between the two groups. MRI wash-out parameters were useful to distinguish both lesions with homogeneous and heterogeneous signal drop from those with no signal loss, even though the accuracy metrics were slightly inferior for lesions with heterogeneous signal drop compared to those with homogeneous signal drop. This latter finding could be due to the presence of different benign, non-adenoma lesions, such as myelolipomas and pheochromocytomas, with heterogeneous signal drop in the same group of AL. These findings suggest that AWO and RWO values do not provide an added value for the characterization of adrenal lesions with heterogeneous CS signal drop. While other quantitative DCE parameters are worthy of investigation, it might be possible to consider an unenhanced MRI protocol to characterize these AL, reducing scanning time and cost while increasing patients’ comfort.

Of note, in our series, all lesions with these CS patterns were benign being mainly (86%) represented by adrenal. Conversely, in AL with no signal drop on CS, the values of wash-out parameters, both of RWO and AWO, were significantly lower, with percentage values of both RWO and AWO suggestive of non-adenomas adrenal tumors, even with the occurrence (33%) of “lipid-poor” adenomas in this group of lesions. Our results are in line with the data reported in the literature regarding the percentage values of RWO and AWO observed in different AL such as adenomas, benign, non-adenomas, and malignant adrenal tumors [13,23,24,25,26,27]. Indeed, a previous study [13] reported that “lipid-poor” adenomas may show RWO and AWO values, both at 5 and 10 min after contrast administration, significantly lower compared to “lipid-rich” adenomas, but similar to those of non-adenoma lesions such as pheochromocytoma or malignant tumors. A reasonable explanation for the low wash-out rates of “lipid-poor” adenomas might be the small amount of fat foci that makes them similar to non-adenoma tumor lesions and determines contrast retention in the extracellular/interstitial space. Hence, the characterization of “lipid-poor” adenomas still remains a diagnostic imaging dilemma. For this purpose, alternative MRI sequence such as T2-weighted, nuclear medicine, or artificial intelligence techniques may be helpful [27,28,29,30,31,32]. An additional interesting finding of our MRI study was that the observed results of RWO and AWO in the three groups (homogeneous, heterogeneous, or no signal drop) of AL occurred both at 5 and 10 min after contrast administration. These data are concordant with the results of other studies which suggested that lesion wash-out may be assessed earlier at 5 min post-contrast injection, shortening the dynamic imaging protocols both on MRI [7] and computed tomography (CT) [17,18,19]. In this regard, a modified MRI protocol using different imaging parameters has been recently proposed for the characterization of adrenal nodules [26]. On a final note, it should be highlighted that DCE quantitative parameters values showed greater ranges than ASII in our study; while we cannot provide a clear explanation to this finding, it might suggest a higher degree of variability, making ASII preferable over RWO and AWO.

Our study has several limitations, the first represented by the retrospective nature of the study itself that influenced lesion grouping. Second, our patient population was also limited in the occurrence of malignant adrenal tumors. Thus, our results need to be confirmed by additional, preferably multicenter, studies. Third, the histological reports were available only in 23 lesions in our series; thus, the overall correlation between imaging and pathology was limited. Fourth, the use of AWO and RWO derived from MRI is less standardized compared to their CT counterpart and might still need further validation studies. Finally, both the qualitative evaluation of CS MR images for lesions grouping and image analysis to obtain the quantitative parameters values were performed working in consensus; therefore, it was not possible to test the reproducibility of measurements; however, the two radiologists reached a perfect agreement in every case without the need of a third evaluator to solve eventual discrepancies, suggesting a good reproducibility.

## 5. Conclusions

AL showing visually heterogeneous CS signal drop may occur in clinical practice on MRI. Although this CS pattern appears to be indicative of the presence of benign AL, mainly represented by adenomas, the occurrence of other benign, non-adenoma lesions is possible. A quantitative CS parameter (ASII) can be effectively used to classify such AL. Quantitative DCE parameters, such as RWO and AWO, show similar values in AL with homogeneous and heterogeneous CS signal drop, confirming their benign nature; however, the accuracy of such DCE parameters was inferior to that of ASII. Of note, ASII seems to be the only quantitative parameter able to differentiate AL among the three different groups.

## Figures and Tables

**Table 1:** Lesion grouping according to qualitative analysis of chemical-shift MRI sequence.

Groups	Qualitative CS Signal Drop	Lesion Type	Total
1	Homogeneous	Adenomas (*n* = 19)	19
2	Heterogeneous	Adenomas (*n* = 17) Pheochromocytomas (*n* = 3) Myelolipomas (*n* = 3)	23
3	Absent	Adenomas (*n* = 10) Pheochromocytomas (*n* = 9) Primary malignant tumors (*n* = 5) Metastasis (*n* = 5) Oncocytoma (*n* = 1)	30

**Table 2:** Quantitative analysis results of adrenal signal intensity index and wash-out MRI parameters in the three groups of lesions.

MRI Parameter	Group 1 Median (Range)	Group 2 Median (Range)	Group 3 Median (Range)
ASII	71 ^#^ (46–87)	53 ^#^ (22–76)	3 ^#^ (−41–75)
AWO_5min_	23 (−49–51)	20 (−133–52)	1 * (−259–45)
AWO_10min_	54 (6–68)	46 (−160–66)	15 * (−372–70)
RWO_5min_	17 (−30–36)	12 (−37–35)	−1 * (−84–31)
RWO_10min_	33 (4–48)	29 (−13–43)	5 * (−100–42)

All values are expressed in percentages. ASII = adrenal signal intensity index, AWO = absolute wash-out, RWO = relative wash-out. ^#^ ASII median values are significantly different in the three groups (*p* < 0.001). * All median values in this group are significantly different from corresponding median values in the remaining two groups (*p* < 0.001).

**Table 3:** Optimal cut-off values and areas under the curve to differentiate adrenal lesions using quantitative MRI parameters.

MRI Parameter	Group 1 and Group 2		Group 1 and Group 3		Group 2 and Group 3	
	Cut-off (95% CI)	AUC (95% CI)	Cut-off (95% CI)	AUC (95% CI)	Cut-off (95% CI)	AUC (95% CI)
ASII	60 (55–62)	0.85 ^#^ (0.71–0.94)	37 (32–37)	0.96 * (0.87–1.00)	20 (7–32)	0.93 ^#^ (0.82–0.98)
AWO_5min_	14 (−36–51)	0.55 (0.38–0.70)	9 (8–25)	0.75 (0.60–0.86)	9 (8–45)	0.70 (0.56–0.82)
AWO_10min_	38 (31–61)	0.61 (0.44–0.75)	36 (35–53)	0.87 (0.74–0.95)	36 (35–53)	0.73 (0.59–0.84)
RWO_5min_	12 (−23–23)	0.54 (0.38–0.70)	6 (3–21)	0.78 (0.64–0.89)	6 (2–24)	0.73 (0.59–0.85)
RWO_10min_	3 (−4–29)	0.63 (0.47–0.78)	23 (18–34)	0.88 (0.75–0.95)	27 (11–34)	0.76 (0.62–0.87)

ASII = adrenal signal intensity index, AWO = absolute wash-out, RWO = relative wash-out, AUC = area under the receiver operating characteristic curve, CI = confidence interval. Cut-off values are expressed as percentages. ^#^ The AUC of ASII is significantly higher compared to AWO_5min_, AWO_10min_, RWO_5min_, and AWO_10min_ (*p* < 0.05). * The AUC of ASII is significantly higher compared to AWO_5min_ and RWO_5min_ (*p* < 0.05).
